# Research Progress of Acoustic Monitoring Technology in Welding and Additive Manufacturing Processes

**DOI:** 10.3390/mi17020246

**Published:** 2026-02-13

**Authors:** Qiang Zhu, Zaile Huang, Huan Li

**Affiliations:** 1 School of Robotics, Guangdong Open University, Guangzhou 510091, China; qzhu@gdpi.edu.cn; 2 School of Mechanical Engineering, Yangtze University, Jingzhou 519016, China; 519016@yangtzeu.edu.cn

**Keywords:** welding, additive manufacturing, structural acoustic emission monitoring, airborne sound monitoring

## Abstract

Continuous innovations in welding and additive manufacturing (AM) technologies have introduced challenges related to process stability and uncertainties in ensuring high quality. Given the high complexity and transient nature of these processes, effective online acoustic monitoring is crucial for ensuring manufacturing quality and improving processing efficiency. This paper first elucidates the principles of acoustic signal generation in welding and additive manufacturing. It then provides a comprehensive review of the application of acoustic methods for quality monitoring in these processes, covering both structural acoustic emission (AE) and airborne acoustic monitoring techniques. Finally, it summarizes the current challenges and issues faced by acoustic monitoring technologies in welding and additive manufacturing and outlines potential future development directions.

## 1. Introduction

Welding has been widely applied across all industrial fields over the past few decades [1,2]. However, numerous uncertainties during the welding process can lead to reduced quality and the occurrence of defects, including the influence of process parameters, workpiece surface conditions, and environmental factors [3,4,5]. Online monitoring systems demonstrate significant advantages in reducing defect rates, enhancing process repeatability, and improving cost efficiency. Consequently, the development of such systems is widely regarded as a key solution to these challenges [6,7].

Additive manufacturing (AM) is an advanced manufacturing method that constructs parts layer by layer using Computer-Aided Design (CAD) models and numerical control systems. By integrating principles from multiple disciplines such as materials science, computer technology, mechanical engineering, and electrical engineering, AM is widely applied in aerospace, defense, shipbuilding, and other fields [8]. It is particularly suited for producing large-scale components from polymers, metals, and ceramics [9]. Compared with traditional subtractive manufacturing, AM offers advantages including the ability to produce complex structures through near-net-shape forming and a high degree of digitalization, leading to its increasing application in recent years [10]. However, a critical issue in AM is its typically open-loop control system, which requires pre-experimental calibration of material and process parameters that are preset as fixed values before manufacturing begins. This lack of adaptive adjustment during execution makes it difficult to address dynamic process variations effectively and increases the likelihood of defect formation [11].

Given the high complexity and transient nature of both welding and additive manufacturing processes, developing effective online monitoring technologies is crucial for ensuring quality and improving efficiency. Process monitoring aims to detect part defects using various methods, including electrical-signal monitoring [12], vision-based monitoring [13], temperature monitoring [14], optical monitoring [15], and acoustic monitoring. For instance, Wang et al. [16] developed a novel Online Surface Height Measurement Model (OSHMM) for Gas Tungsten Arc Welding (GTAW) that integrates electrical signals (welding current, voltage, and arc length) with height displacement data to achieve real-time measurement of surface deposition height. Zhang et al. [13] developed a coaxial visual monitoring system with an auxiliary light source for real-time monitoring of weld width and penetration depth in fiber laser welding, using image processing algorithms to extract weld pool contours. Chen et al. [14] used an infrared camera to capture temperature signals during laser welding, extracting morphological parameters of the molten pool that correlate with welding stability. Among these methods, acoustic monitoring is favored for its non-contact nature, low cost, high information density, rapid response, and ease of sensor setup [17]. Acoustic signals originate directly from physicochemical changes during manufacturing, such as arc fluctuations and molten pool oscillations, propagating instantaneously as sound pressure waves. Acoustic monitoring is particularly effective at detecting phenomena invisible to the naked eye, including micro-porosity in deep-penetration welding, making it a crucial research frontier. However, comprehensive reviews of acoustic applications in these fields remain scarce.

Acoustic monitoring methods primarily include structural acoustic emission (AE) monitoring and airborne acoustic monitoring. AE monitoring evaluates structural health by capturing high-frequency stress waves released from material deformation or damage. Airborne acoustic methods utilize microphones to collect and analyze sound waves propagating through air, offering a simple, non-contact, and cost-effective measurement system. This paper elucidates the acoustic signal generation mechanisms in welding and AM, summarizes applications from both AE and airborne acoustic perspectives, identifies current challenges, and outlines future development directions.

## 2. Principle of Acoustic Signal Generation in Welding and Additive Manufacturing Processes

### 2.1. Acoustic Signal Generation in Welding

**Figure 1:**
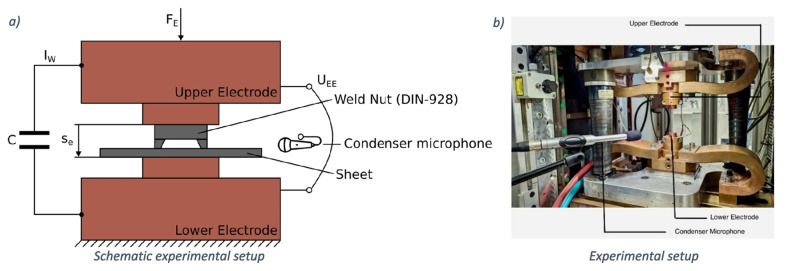
(**a**) Schematical and (**b**) experimental of setup [18].

A schematic of the experimental setup for airborne sound monitoring during resistance spot welding is shown in Figure 1a. A capacitive microphone was positioned horizontally on the mating plane at a distance of approximately 1 m to acquire sound pressure data [18], as illustrated in Figure 1b.

**Figure 2:**
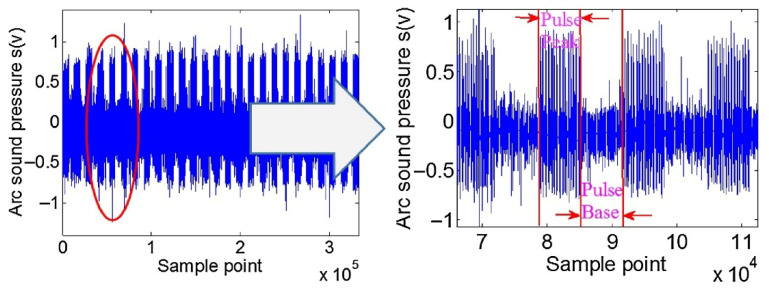
Original audible sound of the welding process [21].

During arc welding, Prezelj et al. [19] experimentally analyzed noise in Gas Metal Arc Welding (GMAW), finding that it primarily consists of pulsed noise and turbulent noise. Pulsed noise originates from arc quenching and re-ignition during short-circuiting transfer. Turbulent noise arises from sources such as the reciprocating oscillations of the arc, electrodes, and molten pool, as well as plastic deformation from internal stress relaxation. Horvat et al. [20] reported a total sound pressure level of 91.5 dB(A), with turbulent noise alone at 81.0 dB(A). Pulsed noise exceeded turbulent noise by 10 dB(A), indicating it can represent the dominant radiated noise mechanism. Lv et al. [21] captured acoustic signals during pulsed GTAW, showing that the original arc acoustic signal consists of process-related signals and ambient noise (Figure 2). This signal exhibits pulsation synchronized with welding current pulses, indicating its generation mechanism is directly related to periodic fluctuations in arc energy.

**Figure 3:**
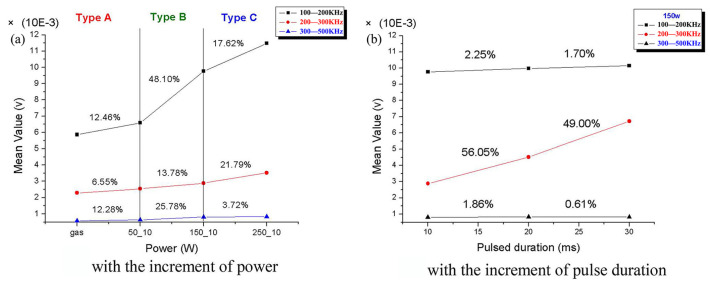
Mean value (**a**) and increasing rates (**b**) for each frequency level [25].

Huang et al. [22] noted that airborne acoustic signals during laser welding mainly originate from pressure variations caused by molten-pool pulsation, metal vapor, thermal stress, and pore oscillation. Grad et al. [23] measured acoustic waves in air and the workpiece during GMAW using microphones and piezoelectric (PZT) sensors, finding that arc-generated sound is primarily caused by short-circuiting and arc re-ignition. Acoustic waves exhibit distinct characteristics under different welding conditions, with arc re-ignition being the main sound source in short-circuit transfer mode. Luo et al. [24] found a strong correlation between structural AE signals detected during pulsed GMAW and grain evolution, indicating the welding arc as the primary vibration source. Increased heat input leads to grain growth and a rise in average AE counts. Lee et al. [25] identified AE sources in laser welding involving rapid liquid–solid phase transitions, plasma formation, and gas pressure from coaxial air jets. Figure 3 shows the average frequency amplitude of acoustic signals against variations in laser power and pulse duration. Increased laser power caused the highest signal amplification in the 100–200 kHz range during welding mode transitions, indicating signals from internal weld structure formation. Significant signal variations in the 200–300 kHz range with pulse duration changes represent molten-pool and heat-affected-zone formation, associated with microstructure evolution and crack initiation.

### 2.2. Acoustic Signal Generation in Additive Manufacturing

**Figure 4:**
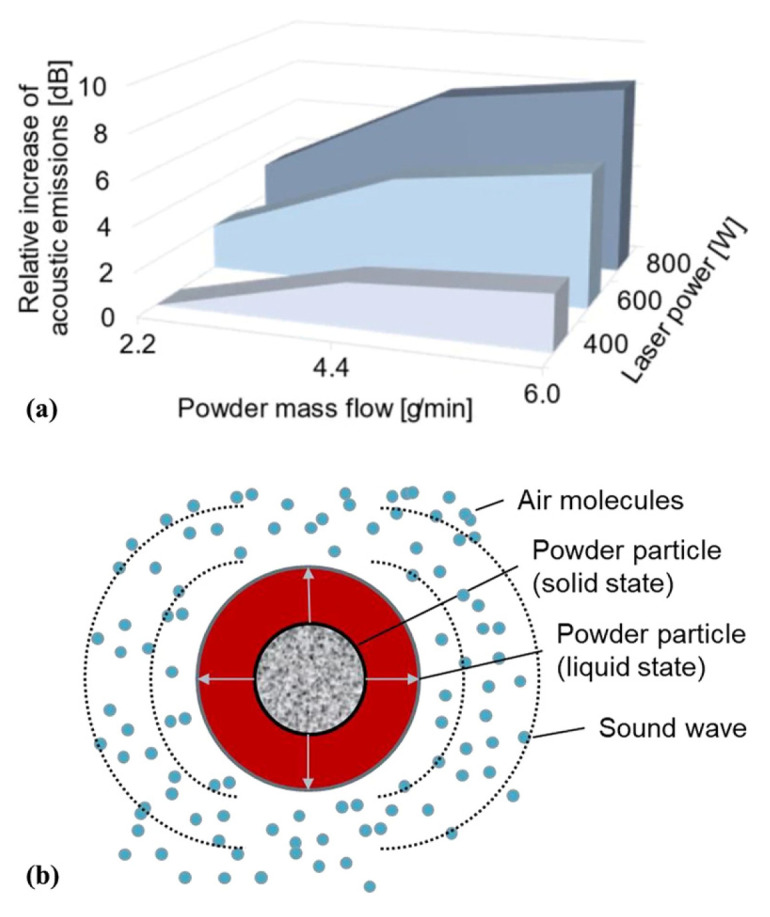
(**a**) Correlation of acoustic emissions with powder mass flow and laser power for LMD processes outside a suitable process window, and (**b**) the possible physical origin of the acoustic emissions in LMD [26].

Directed Energy Deposition (DED) achieves layer-by-layer material deposition by combining powder or wire feedstock with focused energy sources (e.g., lasers, electron beams, or arcs). Both Wire-Arc Additive Manufacturing (WAAM) and Laser Metal Deposition (LMD) are DED variants. Hauser et al. [26] investigated acoustic emission in DED, identifying the expansion of arc plasma as the primary acoustic source in arc-based AM. Acoustic emission anomalies were predominantly associated with arc dimensions. The research also revealed that in laser-based DED, average acoustic emission intensity increases with higher laser power and powder mass flow rates (Figure 4a), indicating sound generation from laser energy transfer to powder particles. Figure 4b illustrates that acoustic emission results from two mechanisms: volume expansion during particle melting and volume contraction during solidification, both of which stimulate the surrounding air.

**Figure 5:**
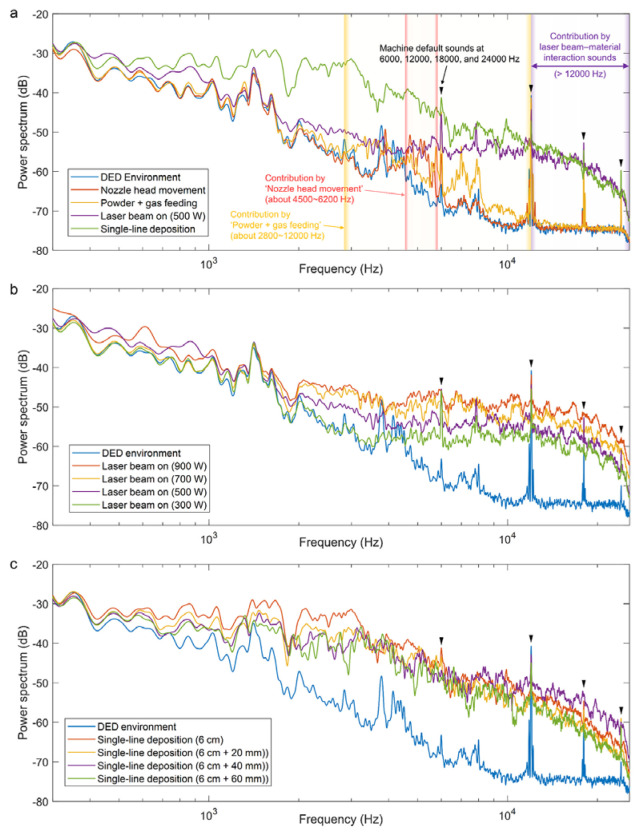
Power spectral density (PSD) plots of acoustic signals captured during the laser metal deposition (LMD) process under three distinct powder feed rate conditions: (**a**) Test #1 (below optimal rate), (**b**) Test #2 (optimal rate), and (**c**) Test #3 (above optimal rate) [27].

Kim et al. [27] found that the power spectrum of sound generated during laser activation (500 W) in Laser Directed Energy Deposition (LDED) is similar to that from single-wire deposition (Figure 5), indicating that the beam–material interaction is the primary source of high-frequency sound. The interaction-induced sound results from the intense metal vapor jet generated when the laser vaporizes the molten pool surface, creating significant friction with the surrounding air.

## 3. Application of Acoustic Methods in Monitoring of Welding Process

### 3.1. Structural Acoustic Emission Monitoring

**Figure 6:**
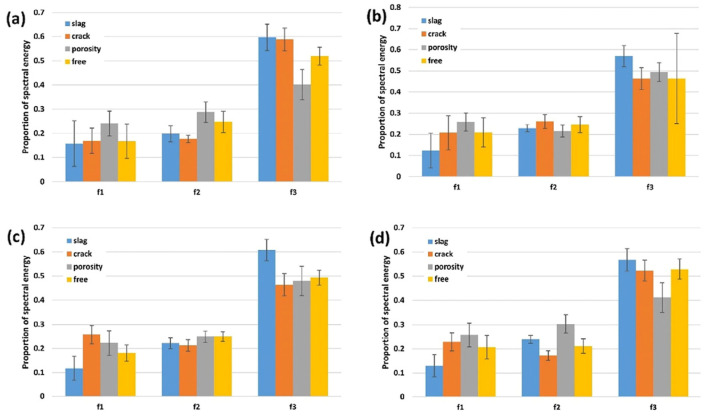
Proportion of spectral AE energy in raw frequency bands for all samples at: (**a**) arrangement 1, (**b**) arrangement 2, (**c**) arrangement 3, and (**d**) arrangement 4 [28].

The acoustic emission (AE) method detects internal defects using signals generated from flaws. Droubi et al. employed AE to detect slag, porosity, and cracks during welding. Frequency analysis using Fast Fourier Transform (FFT) and Wavelet Transform was conducted. The results in Figure 6 indicate that among the measured AE parameters, energy, root mean square (RMS), and peak amplitude were key for defect detection due to their significant percentage differences from defect-free values. Crack defects were most challenging to characterize due to minimal percentage differences from reference values. Differences slightly increased with decreasing sensor–source distance, demonstrating the influence of spatial separation. Wavelet transform accurately detected defective welds and classified defect types, enhancing method sensitivity.

**Figure 7:**
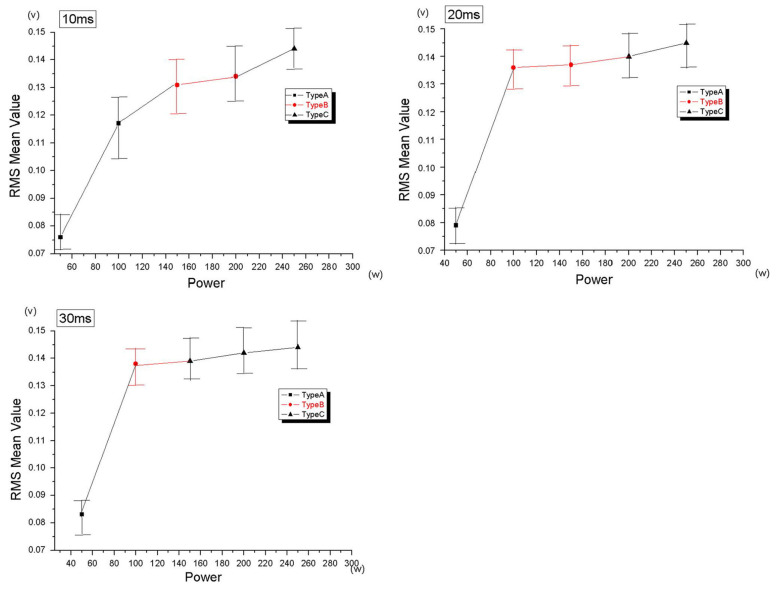
RMS vs. laser power and welding types for each pulse duration [25].

Lee et al. [25] used AE as a feedback signal during laser spot welding (LSW) of stainless steel under different conditions, revealing a significant correlation between AE signals and laser power. Figure 7 shows AE RMS values increasing proportionally with laser power across different pulse durations. AE signals effectively characterized welding outcomes: failure, success, success with thermal defects, and potential crack initiation. Using AE features as inputs for a backpropagation neural network achieved welding type predictions with over 88% accuracy.

**Figure 8:**
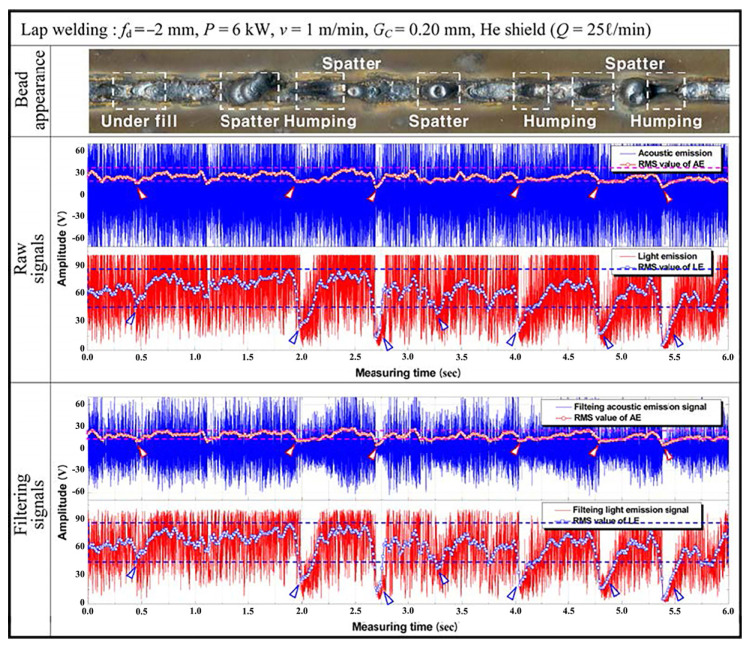
Changes in the raw signals at the 30 μm thick Zn coat and the continuous RMS value by band path filtration [29].

Acoustic methods also detect defects like underfilling and bulging. Lee et al. [29] analyzed acoustic signals during CO_2_ laser lap welding of galvanized steel to investigate the effects of zinc coating thickness and joint gap. Figure 8 shows that within a 0.08–0.2 mm gap range, spatter-related defects correlated with coating thickness, and the acoustic RMS value exhibited significant variations. FFT analysis revealed frequency peaks near 1 kHz for both 15 µm and 30 µm coatings, with amplitude declining as coating thickness increased. Filtering the original signals using FFT results allowed defect identification within the 22 V–38 V range.

### 3.2. Airborne Sound Monitoring

**Figure 9:**
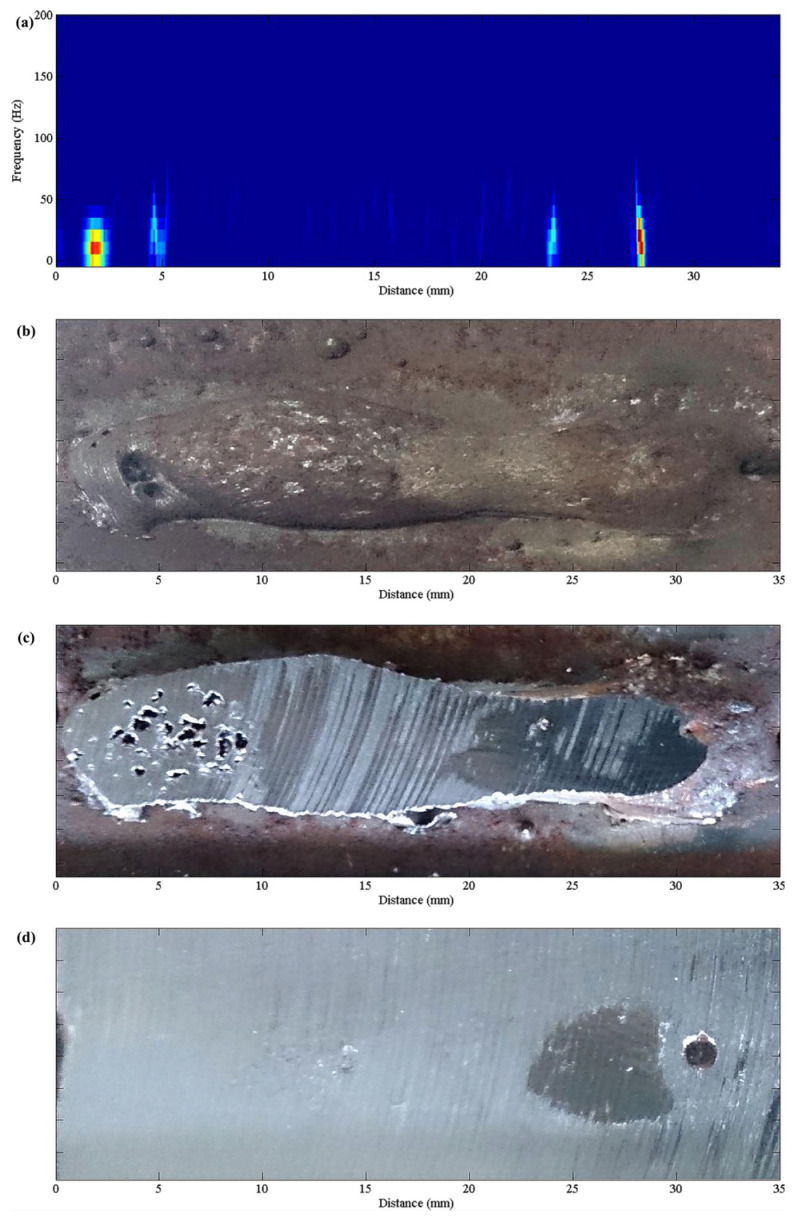
Hilbert–Huang analysis for specimen 1: (**a**) Hilbert spectrum, (**b**) original specimen, (**c**) grounded 2.84 mm depth, and (**d**) grounded 4.15 mm Depth [30].

**Figure 10:**
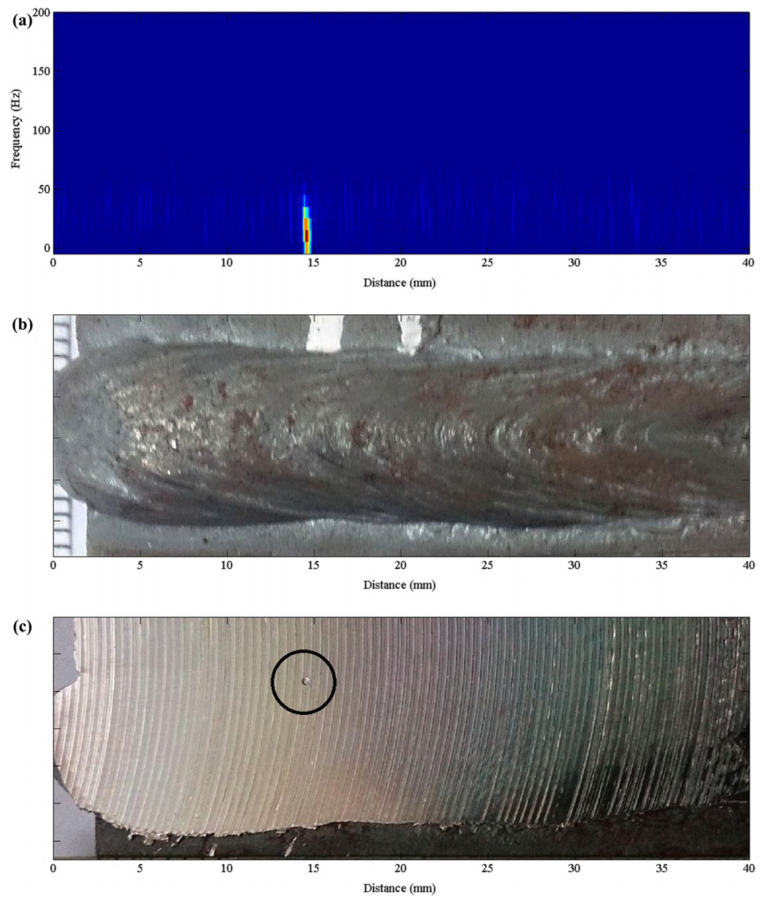
Hilbert–Huang analysis for specimen 2: (**a**) Hilbert spectrum, (**b**) original specimen, and (**c**) grounded 4.87 mm depth [30].

Airborne acoustic methods detect defects such as sub-surface porosity. Yusof et al. [30] used airborne acoustics to detect porosity in API 5L X70 steel during Metal Inert Gas (MIG) welding. Signals were analyzed using the Hilbert–Huang Transform (HHT) to enhance sensitivity. Empirical Mode Decomposition (EMD) decomposed signals into Intrinsic Mode Functions (IMFs). Figure 9 and Figure 10 present Hilbert spectra of IMFs from two samples, showing increased energy amplitude at locations corresponding to sub-surface porosity (e.g., 2–5 mm, ~23 mm, and ~27 mm from the start). Grinding confirmed porosity at depths of 2.84 mm and 4.15 mm, demonstrating HHT’s effectiveness.

**Figure 11:**
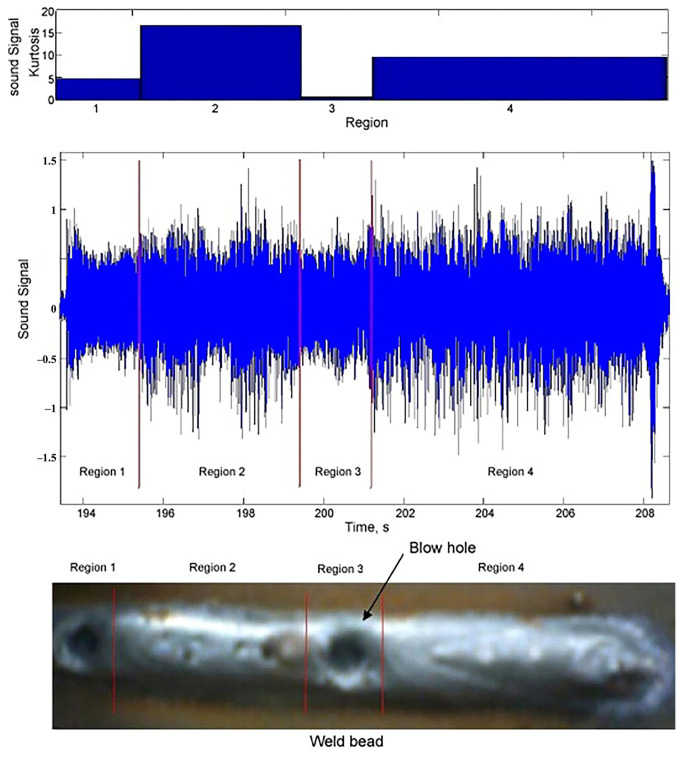
Welding defect monitoring with sound kurtosis [31].

In GMAW, arc acoustic signals closely relate to process parameters and quality. Pal et al. [31] analyzed welding acoustic signals in the time and frequency domains, correlating them with parameters and metal transfer modes. The RMS and kurtosis of arc acoustics characterize droplet transfer stability. High RMS indicates intense transfer (e.g., large droplets with spatter), while low kurtosis indicates stable one-droplet-per-pulse (ODPP) transfer. In an experiment with poor wire condition (Figure 11), significant porosity was identified through changes in arc acoustic kurtosis. The defective weld pool region showed no metal transfer and lower kurtosis variation, with wire corrosion causing instantaneous ablation and extremely low kurtosis, confirming kurtosis as a superior indicator for porosity.

**Figure 12:**
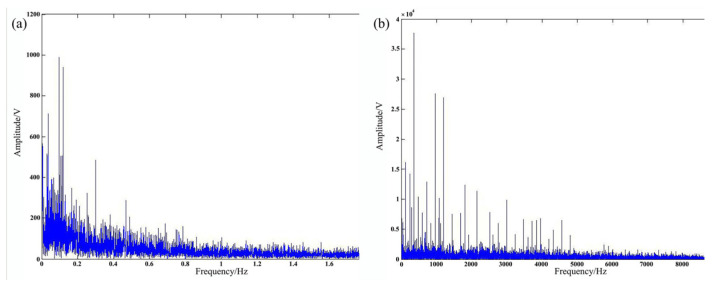
Frequency feature of (**a**) the defect acoustic signal and (**b**) the normal laser acoustic signal [32].

Sansan et al. [32] developed a blind source separation technique integrating Principal Component Analysis (PCA) and Independent Component Analysis (ICA) to capture distinct acoustic signals from micro-pores during laser welding. The analysis successfully distinguished acoustic signatures between normal welds and perforation defects (Figure 12), decomposing signals into cooling-related and keyhole components.

**Figure 13:**
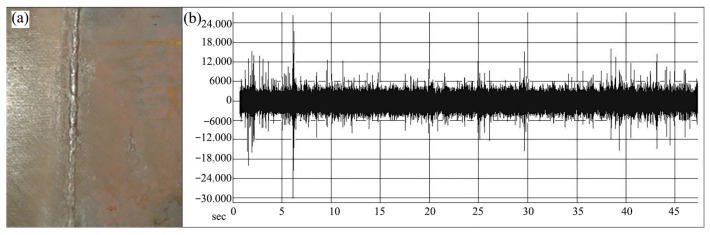
(**a**) Good weld specimen; (**b**) good-weld sound signal [33].

**Figure 14:**
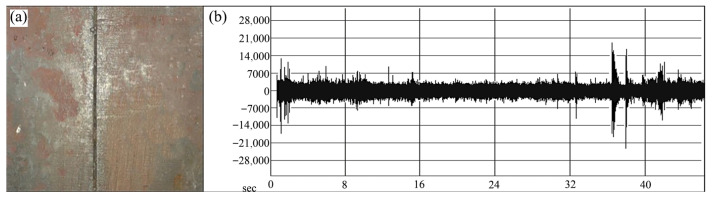
(**a**) Joint with lack of fusion; (**b**) lack-of-fusion sound signal [33].

**Figure 15:**
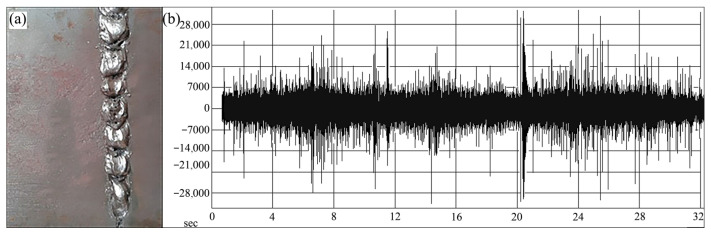
(**a**) Joint with burn-through (**b**) burn-through sound signal [33].

Airborne acoustic monitoring can also be applied to weld quality classification. Sumesh et al. [33] conducted studies on manual-metal-arc (MMA) welding of carbon steel plates and captured associated welding arc sounds. Statistical features were extracted from the acoustic signals and used as inputs for two machine learning classifiers: J48, a widely adopted implementation of the decision tree algorithm known for producing transparent, rule-based models, and the Random Forest algorithm. Both methods successfully categorized welds into three distinct classes: high-quality welds (Figure 13), incomplete fusion (Figure 14), and burn-through (Figure 15). The Random Forest classifier achieved an accuracy of 88.69%, outperforming the J48 classifier, which attained 70.78%. These results indicate that acoustic signatures effectively reflect underlying welding conditions and demonstrate the viability of acoustic-based methods for automated quality control.

**Figure 16:**
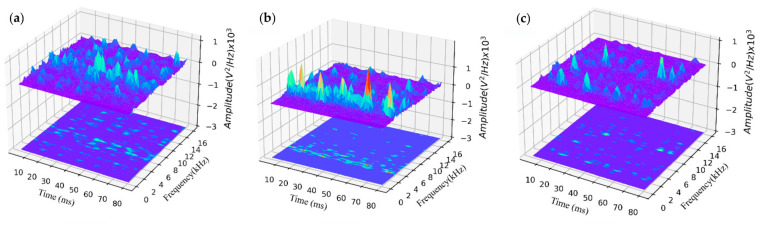
Time–frequency spectrum of different welding states. (**a**) Normal. (**b**) Burn-through. (**c**) Lack of fusion [34].

Yang et al. [34] investigated acoustic mechanisms in cold-metal-transfer (CMT) welding via time-frequency analysis. Alternating arc power generates distinct acoustic patterns. Wire reciprocation alters arc length and geometry, and entry into the molten pool induces vibrations. Figure 16b reveals multiple peak amplitudes in the 2–6 kHz range attributable to burn-through and discontinuous burn-through from increased weld gaps. Amplitudes in non-welded zones were lower.

**Figure 17:**
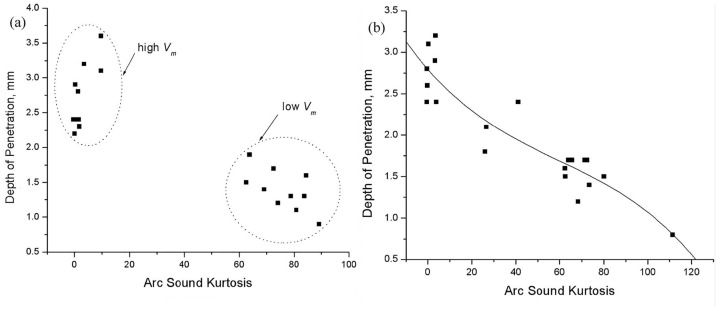
Relationship of arc sound kurtosis with weld penetration for various (**a**) pulse frequencies and (**b**) pulse duty factors [35].

Pal et al. [35] monitored penetration depth in Pulsed-MIG (P-MIG) welding via airborne acoustics, finding a correlation between acoustic signals and depth. Acoustic kurtosis, arc power, and peak temperature were effective for monitoring, with kurtosis performing best. Figure 17a shows penetration depth versus acoustic kurtosis under variable pulse frequency, with data clustering between high and low average voltage conditions. Figure 17b shows enhanced correlation when adjusting the pulse duty cycle.

**Figure 18:**
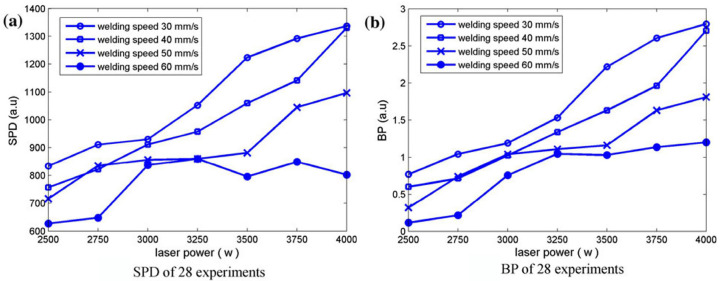
Acoustic signatures of 28 experiments of (**a**) SPD, and (**b**) BP [36].

Huang et al. [36] investigated the relationship between penetration depth and acoustic signals during laser welding of high-strength steel. Signals were preprocessed via spectral subtraction, then analyzed in the time and frequency domains using Sound Pressure Deviation (SPD) and Band Power (BP). Figure 18 illustrates acquired acoustic characteristics. SPD and BP strongly correlate with penetration modes and depth, increasing during the transition from conduction to keyhole mode due to enhanced plasma and metal vapor production.

**Figure 19:**
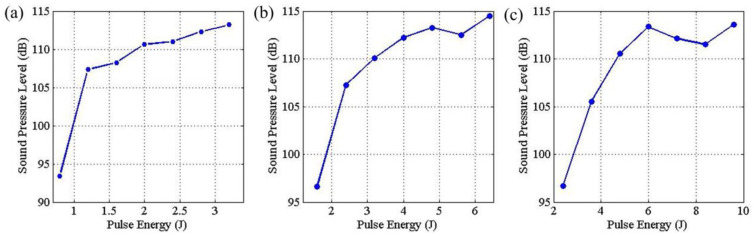
Relation between laser pulse energy and sound pressure level: (**a**) Pulse width of 2 ms, (**b**) pulse width of 4 ms, (**c**) pulse width of 6 ms [37].

Yusof et al. [37] studied the correlation between acoustic characteristics and penetration in pulsed-laser welding. Figure 19 shows that sound pressure levels at a 2 ms pulse width exhibit a linear relationship with pulse energy and penetration depth up to a threshold, beyond which nonlinear behavior is observed.

### 3.3. Summary and Progress in Welding Process Monitoring

Acoustic monitoring has proven to be a highly effective and versatile tool for the in situ assessment of welding processes. Research demonstrates robust correlations between specific acoustic features and critical weld qualities. Structural AE monitoring, through parameters such as energy, RMS, and peak amplitude, reliably detects internal defects like porosity, slag, and cracks. Airborne acoustic monitoring successfully links signal properties (e.g., RMS, kurtosis, spectral peaks) to process stability, transfer modes, penetration depth, and surface/sub-surface defects. A significant advancement is the integration of acoustic signals with advanced signal-processing techniques—including FFT, Wavelet Transform, HHT, and blind-source-separation methods like PCA-ICA—which enhance defect sensitivity and identification. Furthermore, applying machine learning algorithms (e.g., Artificial Neural Networks, J48, Random Forest) to acoustic features has enabled automated weld quality classification with high accuracy. This convergence of sensing, signal processing, and data analytics marks substantial progress toward intelligent, real-time welding process control and quality assurance.

## 4. Application of Acoustic Methods in Monitoring Additive Manufacturing Processes

### 4.1. Structural Acoustic-Emission Monitoring

AE signals are elastic waves generated by rapid energy release from stress redistribution. Piezoelectric sensors on the workpiece surface detect these waves for parametric analysis [38], constituting a passive, non-destructive testing method.

**Figure 20:**
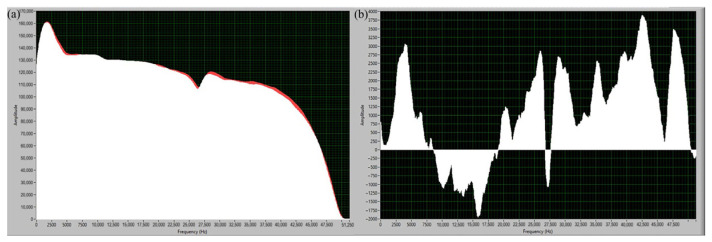
Energy content of optimal and non-optimal scanning over frequency range (**a**). The energy difference between optimal and non-optimal scanning (**b**) [39].

Kouprianoff et al. [39] employed AE for online monitoring of lack-of-fusion and spheroidization in single-pass Powder Bed Fusion (PBF) of maraging steel, using Short-Time Fourier Transform (STFT) for analysis. Figure 20 illustrates amplitude differences between optimal and suboptimal scanning signals, with significant variations in the high-frequency range serving as an indicator for porosity or under-melting.

**Figure 21:**
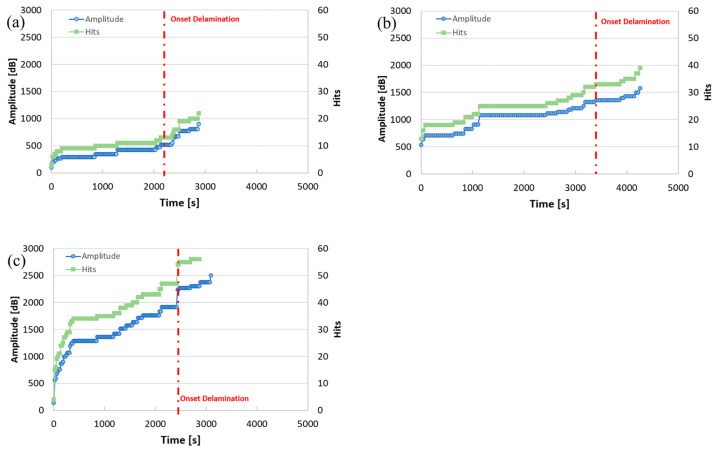
Acoustic emissions recorded: (**a**) refers to Group A specimen (220 °C); (**b**) refers to Group B specimen (230 °C); (**c**) refers to Group C specimen (240 °C) [40].

Barile et al. [40] investigated the effect of extrusion temperature on interlayer cohesion in Fused-Deposition Modeling (FDM) of ABS. Mechanical tests on double-cantilever beam (DCB) specimens with AE recording showed the highest amplitude in Group C samples (Figure 21), correlating with the highest temperature and increased shear toughness. AE detected delamination onset via increased impact frequency and amplitude seconds before failure.

**Figure 22:**
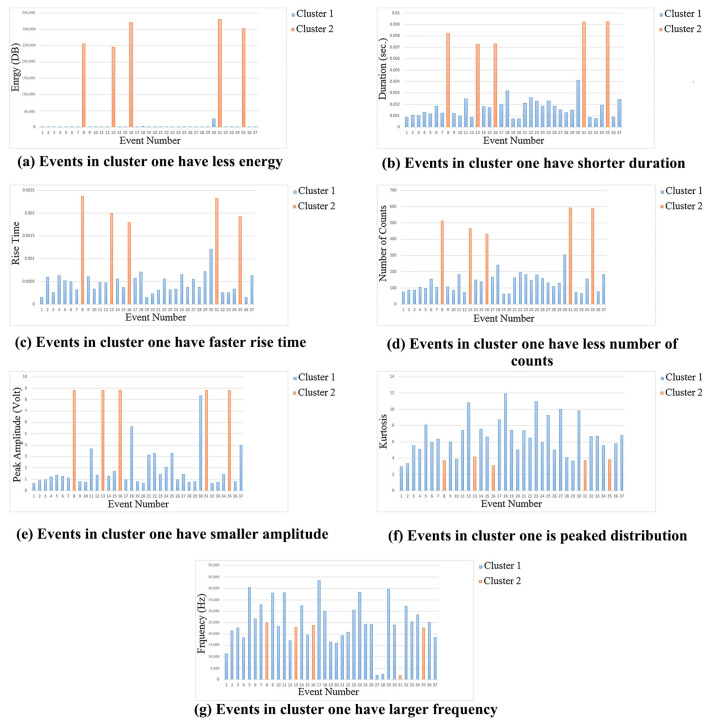
(**a**–**g**) Comparing the features of the AE events in clusters one and two [41].

Gaja et al. [41] used AE sensors to monitor laser deposition. Cluster analysis of AE data correlated results with defect sources in LMD. As shown in Figure 22, pore-related AE events exhibited higher energy, shorter decay time, and smaller amplitude than crack-related events, with signal energy being the key discriminator.

**Figure 23:**
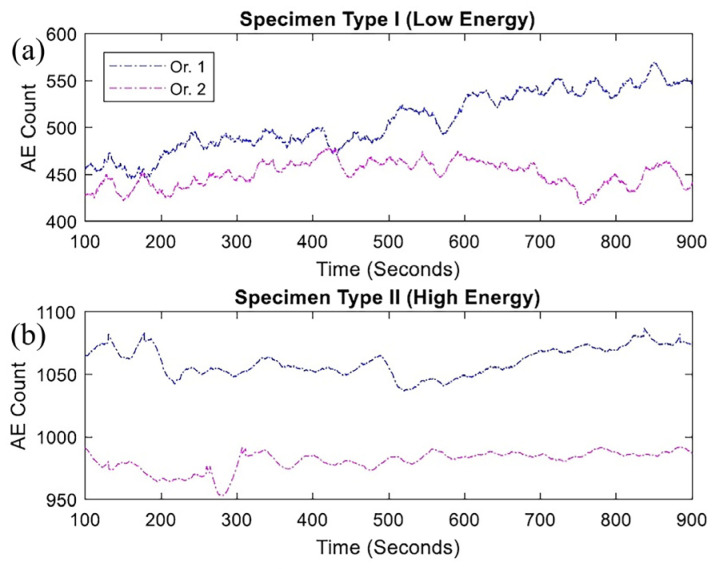
AE count for type I (**a**) and type II (**b**) specimens in two orientations [42].

**Figure 24:**
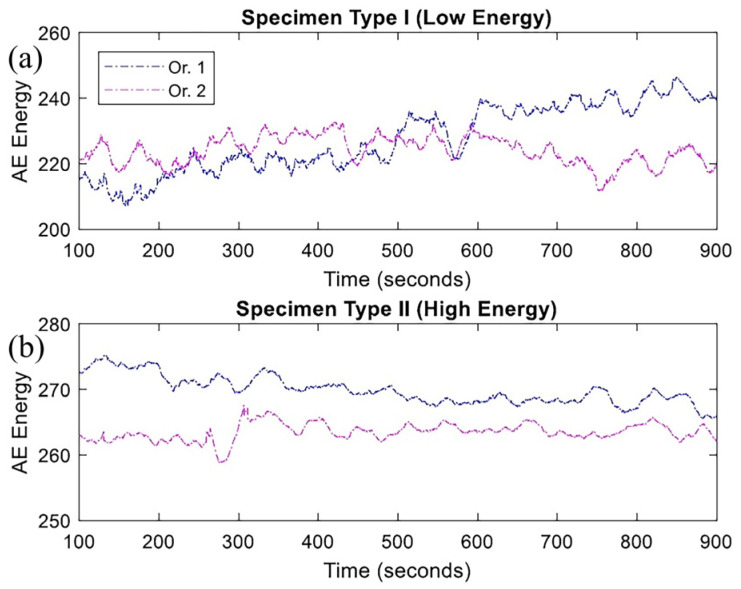
AE energy (10 μVs/count) for type I (**a**) and type II (**b**) specimens in two orientations [42].

Niknam et al. [42] applied AE to analyze anisotropy in Ti-6Al-4V fabricated via DED under three-point-bending cyclic loading. Figure 23 and Figure 24 show AE counts and energy for samples produced with different laser powers. Higher laser power resulted in higher AE counts and energy. For low-power samples, AE characteristics were similar across test directions; high-power samples showed stronger AE signals along the build direction.

### 4.2. Airborne Acoustic Monitoring

**Figure 25:**
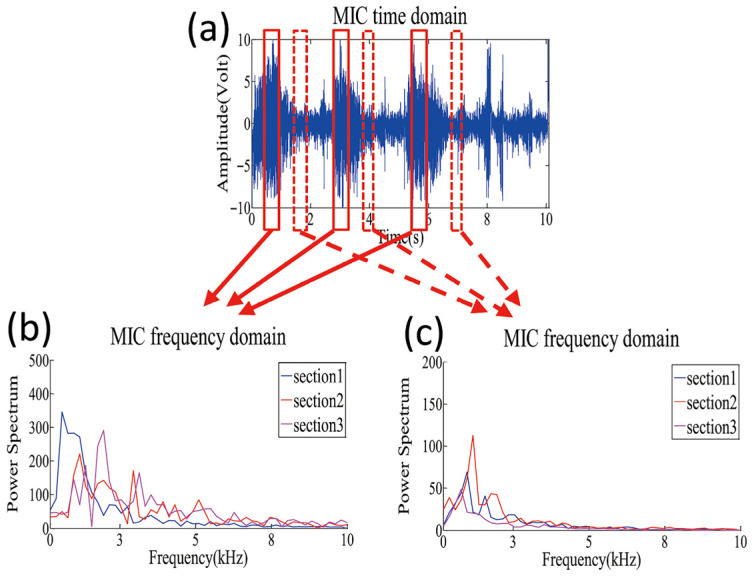
Frequency responses during deposition without the generation of balling defects, during the generation of balling defects, and at the start of deposition: (**a**) Time domain signal obtained under the generation of balling defects, (**b**) power spectra obtained under the generation of balling defects and at the start of deposition, and (**c**) power spectrum obtained under deposition without the generation of balling defects. These experimental results were obtained at GeniRay Technology Corporation, New Taipei City, Taiwan, China [43].

Low-cost, easily installed microphones can detect molten-pool vibrations and geometric changes. Wu et al. [43] conducted DED on stainless steel using Inconel 718, collecting audible acoustic signals with MEMS microphones. They studied the relationship between deposition dimensions, spheroid defects, and acoustic signals. Changes in liquid-metal geometry and deposition point shifts triggered spheroid defects. Acoustic analysis confirmed a strong correlation between audible characteristics and melt volume, with RMS amplitude correlating with deposited-spheroid size and signal energy increasing with molten volume. Figure 25 shows frequency responses during initial deposition, defect-free deposition, and spheroid defect presence. Initial deposition energy concentrated below 1.5 kHz; spheroid defect formation clustered at higher frequencies. Defect-free deposition concentrated in the 1–3 kHz range with lower energy.

**Figure 26:**
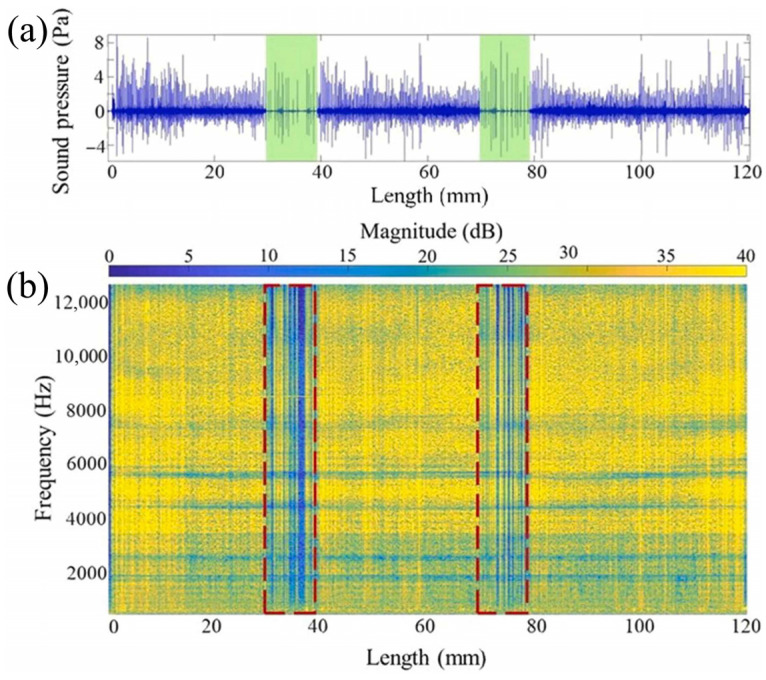
Acoustic signal obtained during deposition of layer #7 of sample C1. (**a**) Time domain representation. (**b**) Time–frequency domain representation. The green regions in (**a**) indicate where perturbation of the sound pressures occurs, which correspond to the areas outlined by the red boxes [44].

**Figure 27:**
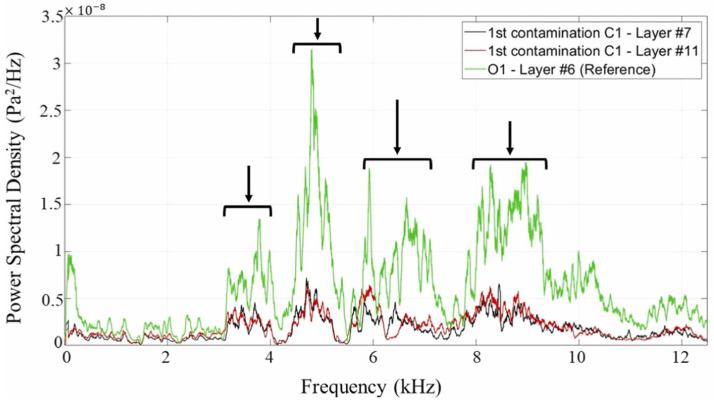
A Power Spectral Density (PSD) plot for a 1 s interval inside the contaminated layers #7 and #11 from sample C1 and layer 6 from sample C1, taken as a reference. The black arrows indicate the frequencies that differ greatly from the reference signal [44].

To characterize pollutant effects on WAAM, Ramalho et al. [44] used microphone-based acoustic sensing on parts contaminated with chalk, oil, and sand. Acoustic signals were analyzed via time domain analysis, Power Spectral Density (PSD), and STFT. Figure 26b shows the STFT of an acoustic signal from a chalk-defective sample, with contaminated areas showing transient acoustic peaks interspersed with low-intensity intervals. The red box indicates the location of the acoustic pressure disturbance. PSD applied to 1 s intervals within contaminated regions (Figure 27) showed intensity below the reference at most frequencies, aiding defect identification.

**Figure 28:**
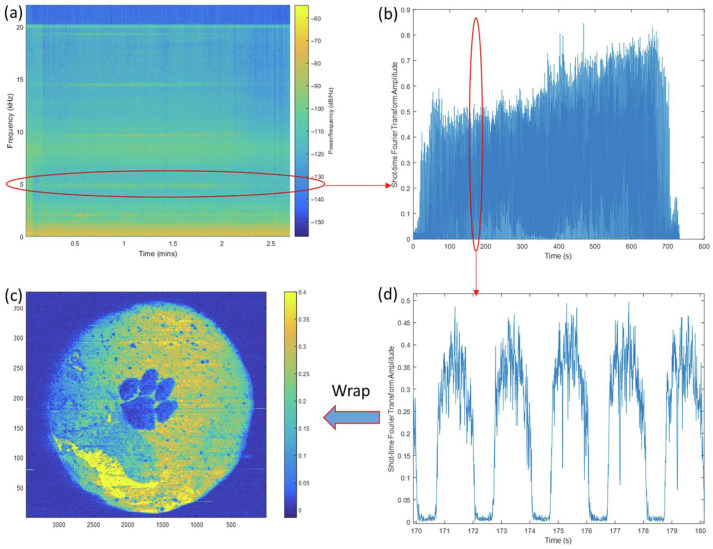
General signal processing steps in obtaining the PA image. (**a**) Spectrogram of a complete sound data set when scanning a layer of the workpiece. (**b**) Extracting only the data of 5 kHz and plotting it on the time axis. (**d**) Zoomed-in view of a small section of (**b**). (**c**) Wrap up the signal in (**b**) according to laser scan position data, and the PA image is obtained [45].

Cheng et al. [45] captured photoacoustic waves during Laser-Assisted Ceramic Additive Manufacturing (LAMC). STFT generated spectrograms; photoacoustic signals extracted at the laser modulation frequency were combined with laser scan position to obtain images of processed layers (Figure 28), validating detection of metal and dry paste defects.

Gutknecht et al. [46] used a microphone, a coaxial dual-color thermometer, and an anisotropic thermal imager, finding that the microphone showed the highest sensitivity to process deviations.

**Figure 29:**
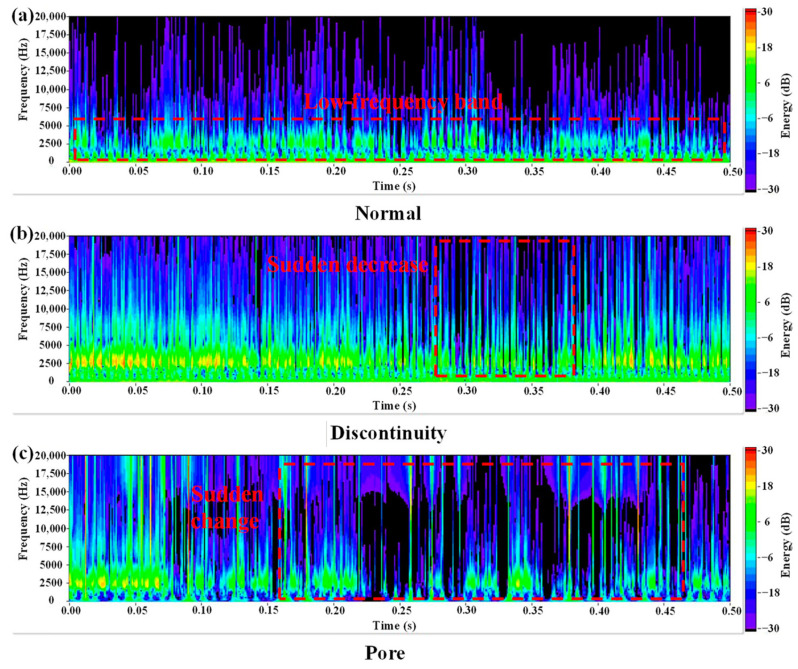
Time–frequency diagrams of acoustic signals: (**a**) Normal, (**b**) discontinuity, and (**c**) pore [47].

Zhang et al. [47] proposed an acoustic-based defect detection method for DED-arc using wavelet time–frequency analysis. Continuous Wavelet Transform converted 1D acoustic signals into 2D time–frequency spectra for Convolutional Neural Network (CNN) training, achieving over 96% accuracy with four CNN models. Figure 29 shows typical time–frequency spectra of normal, discontinuous, and porous welds, with abnormal signals exhibiting greater instability.

**Figure 30:**
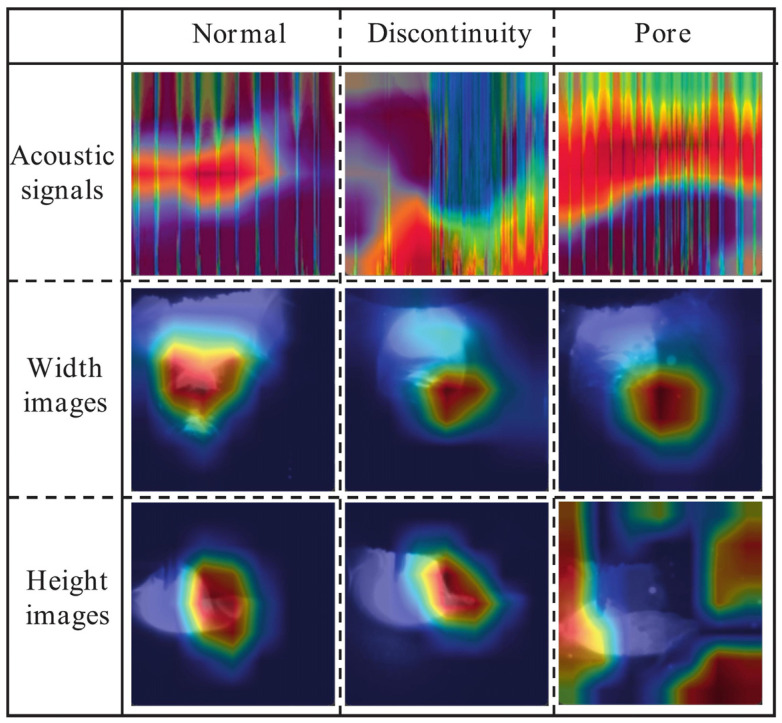
The CAMs of the three signals [48].

Single-sensor systems are susceptible to ambient noise and limited perspective. Zhang et al. [48] developed an in situ defect detection system integrating acoustic signals with molten-pool width and height images via multi-sensor fusion. Three complementary signals from DED-arc identified discontinuities and porosity. Figure 30 shows Class Activation Mapping (CAM) results for pool width, height, and acoustic time–frequency spectra. CNN models showed superior sensitivity to frequency domain variations. Multi-sensor configurations outperformed single-sensor models, with data-level and decision-level fusion achieving 100% and 99.55% accuracy, respectively.

### 4.3. Summary and Progress in Additive Manufacturing Process Monitoring

Acoustic monitoring shows significant promise for process interrogation and defect detection in AM. Structural AE is sensitive to intrinsic energy release events and has successfully identified defects such as lack of fusion, porosity, and delamination in PBF, DED, and FDM processes. AE parameters (counts, energy, amplitude) also provide insights into material anisotropy and the effects of parameters like laser power. Concurrently, airborne acoustic monitoring using low-cost microphones has demonstrated high sensitivity to process deviations, molten–pool oscillations, and geometric defects like balling. A key trend is the move beyond single-sensor analysis. The fusion of acoustic data with complementary signals (e.g., visual, thermal) coupled with advanced time–frequency analysis (STFT, Wavelet Transform) and deep learning models (CNNs) has dramatically improved defect identification accuracy and robustness. This multi-sensor, data-driven approach represents a crucial step toward reliable in situ monitoring systems for closed-loop feedback control in AM.

## 5. Conclusions and Prospects

This review has systematically examined the application of acoustic monitoring technologies, encompassing both structural AE and airborne acoustic sensing, in welding and additive manufacturing. The findings underscore the significant potential of acoustic methods for real-time, non-contact, and information-rich process interrogation. The principal conclusions, prevailing challenges, and prospective research directions are synthesized as follows.

(1) Acoustic monitoring has been conclusively demonstrated to be an effective proxy for a wide range of welding phenomena and defect states. Key correlations have been firmly established. In defect detection, AE parameters (energy, RMS, counts) and airborne acoustic features (kurtosis, spectral peaks) reliably indicate defects including porosity, cracks, lack of fusion, and spatter. For process characterization, airborne sound signals exhibit high sensitivity to metal transfer modes, arc stability, and penetration depth. Effective utilization requires advanced signal-processing techniques (FFT, Wavelet Transform, HHT, PCA-ICA) for feature extraction. Furthermore, integrating machine learning algorithms (ANN, Random Forest, SVM) has enabled automated weld quality classification with high accuracy, marking a significant step toward intelligent process monitoring.
(2) The application of acoustic monitoring in AM is rapidly evolving, demonstrating significant potential for in situ process interrogation and quality assurance. AE monitoring effectively detects critical flaws such as a lack of fusion, porosity, delamination, and balling defects, with signal energy and frequency content serving as critical discriminators. AE activity also correlates with process parameters (e.g., laser power) and microstructural characteristics (e.g., anisotropy). A prominent trend is the move towards multi-sensor data fusion, synergistically combining airborne acoustics with complementary optical or thermal data and analyzing them via deep learning architectures like CNNs. This integrated approach has achieved superior defect detection rates, exceeding 96%, highlighting a pathway toward robust in-process monitoring for AM.

Previous research on acoustic monitoring in welding focused on various processes including LSW, GMAW, MIG, P-MIG, CMT, and MMA. Structural AE monitoring detects internal defects and characterizes process features. Airborne acoustic monitoring correlates arc signals with quality, monitors penetration depth, detects defects, and classifies weld quality into categories such as high-quality, incomplete fusion, and burn-through.

To transition from promising research to reliable industrial technology, future efforts should prioritize:

(1) Mitigating ambient-noise interference in welding acoustic monitoring. Future research should develop advanced time–frequency analysis or other techniques for noise suppression. Current systems are often inadequate; integrated systems combining high-speed cameras, infrared imaging, and acoustic sensors could enable comprehensive quality evaluation through multi-sensor data fusion.
(2) Exploring acoustic monitoring in solid-state welding processes such as Friction Stir Welding (FSW) and Ultrasonic Welding (USW). These processes involve dynamic interfacial interactions (friction, deformation, bonding) that generate rich acoustic signals. In FSW, AE sensing could enable real-time detection of tool wear, volumetric defects, and microstructural changes. The primary challenge is distinguishing defect-related AE from high background noise. For USW, where acoustic energy is intrinsic, passive acoustic monitoring offers potential for quality assessment by analyzing harmonic responses and damping characteristics to evaluate bond quality and detect interfacial flaws.
(3) Integrating Artificial Intelligence (AI) into acoustic monitoring to redefine process intelligence. Key research directions include: leveraging deep learning models (CNNs, RNNs) to autonomously extract features from raw signals; developing multimodal fusion architectures combining acoustic, visual, and thermal data; employing unsupervised/semi-supervised learning to detect novel anomalies; enhancing interpretability via Explainable AI (XAI); and implementing lightweight edge AI systems for real-time adaptive control, advancing toward autonomous, self-optimizing manufacturing.

## Data Availability

No new data were created or analyzed in this study. Data sharing is not applicable to this article.
